# Protocol for a nested case-control study: identifying neuroimaging biomarkers for the progression of subclinical depression and qi-stagnation constitution to major depressive disorder in adolescents

**DOI:** 10.3389/fpsyt.2024.1516846

**Published:** 2025-01-21

**Authors:** Jing Wang, Chengfeng Zhang, Yueqi Zhang, Yuanyuan Liu, Jingli Zhang, Xingwei Fang, Wangyang Xia, Yanzhao Xie, Zhongli Lan, Jinhui Wang, Min Lu, Jun Chen

**Affiliations:** ^1^ Institute for Brain Research and Rehabilitation, South China Normal University, Guangzhou, China; ^2^ Department of Radiology, Guangdong Provincial Hospital of Chinese Medicine, Zhuhai, China; ^3^ Department of Psychiatry, Guangdong Provincial Hospital of Chinese Medicine, Zhuhai, China; ^4^ Department of Traditional Therapy, Guangdong Provincial Hospital of Chinese Medicine, Zhuhai, China; ^5^ Department of Prevention and Health Care, Guangdong Provincial Hospital of Chinese Medicine, Zhuhai, China; ^6^ Department of Information, Guangdong Provincial Hospital of Chinese Medicine, Zhuhai, China; ^7^ The Second Clinical College, Guangzhou University of Chinese Medicine, Guangzhou, China; ^8^ Key Laboratory of Brain, Cognition and Education Sciences, Ministry of Education, Guangzhou, China; ^9^ Center for Studies of Psychological Application, South China Normal University, Guangzhou, China; ^10^ Guangdong Key Laboratory of Mental Health and Cognitive Science, South China Normal University, Guangzhou, China; ^11^ Department of Hospital Office, Guangdong Provincial Hospital of Chinese Medicine, Guangzhou, China; ^12^ Department of Radiology, Guangdong Provincial Hospital of Chinese Medicine, Guangzhou, China

**Keywords:** major depressive disorder, subclinical depression, qi-stagnation constitution, functional magnetic resonance imaging, neuroimaging biomarkers

## Abstract

**Background:**

Major depressive disorder (MDD) frequently results in suboptimal treatment outcomes and elevated recurrence rate, with patients frequently engaging in self-harm and suicidal behavior, thereby placing a heavy burden on families and society. Specifically, MDD in adolescents is linked to an elevated suicide risk. Thus, early identification and intervention is crucial for adolescents at high risk for developing MDD. Subclinical depression (SD), characterized by depressive symptoms that do not meet the full criteria for MDD, substantially increases the risk of developing MDD. According to Traditional Chinese Medicine body constitution theory, Qi-stagnation constitution (QSC) is also considered a significant risk factor for the progression to MDD. This study protocol aims to identify neuroimaging biomarkers for the progression from adolescents with SD and QSC to those with MDD, facilitating early intervention strategies.

**Methods and analysis:**

This nested case-control study includes both longitudinal follow-up and cross-sectional comparison. Three hundred first-year senior high school students diagnosed with SD and QSC will be recruited. The 300 adolescents will undergo rs-fMRI scans at baseline and again after one year. We then divide the 300 adolescents with SD and QSC into two groups based on whether they progress to MDD after one year. Functional brain networks will be constructed based on 400 regions of interest (ROIs). Neuroimaging measures, including regional homogeneity and low-frequency fluctuation for each ROI, as well as graph-based global efficiency, nodal efficiency, and nodal centrality from the binary networks, will then be calculated. Finally, differences in these neuroimaging measures between the two groups at baseline will be analyzed to identify biomarkers that can predict the progression from adolescents with SD and QSC to those with MDD.

**Study registration:**

This study protocol does not involve clinical interventions and is classified as an observational study, so it was not subject to prior registration.

## Introduction

Major depressive disorder (MDD) often yields poor treatment outcomes and high recurrence rates (1, 2), with patients frequently engaging in self-harm and suicidal behavior (3, 4), thereby placing a heavy burden on families and society. Specifically, adolescent MDD correlates with a higher risk of suicide (5). In addition, adolescence is a time of significant neuroplasticity, marked by profound developmental transformations and crucial changes in the brain (6). Previous studies have shown that a large proportion of adolescents with symptoms of depression and substantial distress or dysfunction fail to meet the diagnostic criteria for MDD (7, 8). Therefore, it is crucial to perform early identification and intervention in adolescents at high risk for developing MDD.

Subclinical depression (SD), characterized by depressive symptoms that do not meet the diagnostic criteria for MDD (9), substantially increases the risk of developing MDD (10, 11). Research has found that approximately 12% of adolescents suffering from SD develop MDD (12). In addition, according to Traditional Chinese Medicine (TCM) body constitution theory, different body constitution characteristics are closely linked to the onset of specific diseases, with individual body constitutions affecting susceptibility to pathogenic factors and diseases (13). For example, adult women with Yang-and Yin-deficient constitutions have a higher risk of developing MDD (14), while college students with Qi-stagnation constitution (QSC) and Qi-deficiency constitution (QDC) are more prone to MDD (15). Notably, QSC, characterized by long-term emotional dysfunction and stagnation of Qi movement, is found to be closely associated with MDD in adolescents, as demonstrated by a cross-sectional study that adolescents with QSC have a 3.6 times higher risk of developing MDD compared to adolescents without QSC (16). Thus, both SD and QSC are important risk indicators for developing MDD in adolescents, and in this study, we use these two high-risk factors as key criteria for selecting adolescent participants.

Neuroimaging techniques have been widely used to detect the brain abnormalities in MDD, and further explore their relationships with clinical symptoms of patients (17, 18). Among the techniques, resting-state functional magnetic resonance imaging (rs-fMRI) is notable for its favorable results in terms of reproducibility and consistency across studies (19). Previous studies have applied amplitude of low-frequency fluctuation (ALFF) and regional homogeneity (ReHo) analyses to detect the abnormality of neuronal activity in adolescents with MDD (20, 21). Meanwhile, considerable evidence suggests that examining functional connectivity (FC) of brain regions in adolescents with MDD may provide insight into the etiology of the disorder, particularly in the context of brain changes that occur during this sensitive developmental period (22, 23). Moreover, adolescents with MDD are found to exhibit disruptions in the topological organization of functional brain networks through the graph theoretical analysis of rs-fMRI (24). We anticipate that these abnormal neuroimaging-based measures in adolescents with MDD may also serve as predictive biomarkers for the progression from adolescents with SD and QSC to those with MDD.

In this study, we will enroll 300 first-year senior high school students diagnosed with SD and QSC. All participants will undergo rs-fMRI scans at baseline and again after one year. The adolescents will be divided into two groups based on whether they progress to MDD after one year. We will compare differences between the two groups at baseline, focusing on brain rs-fMRI-derived metrics such as ALFF, ReHo, FC, and graph-based network parameters, to explore neuroimaging biomarkers for the progression from adolescents with SD and QSC to those with MDD.

## Methods and analysis

### Design

**Figure 1:**
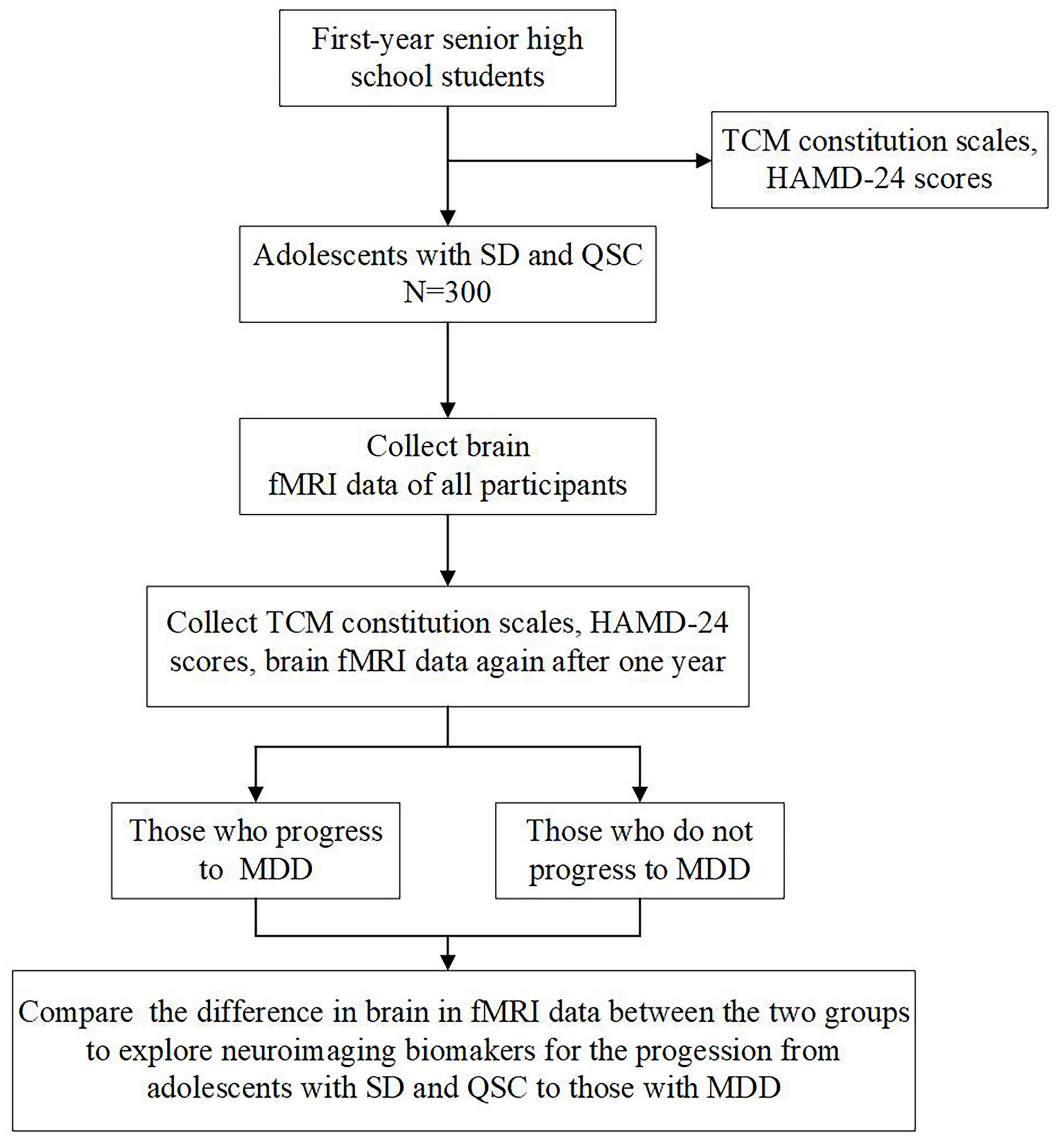
Study flow chart. TCM, Traditional Chinese Medicine; HAMD-24, Hamilton Depression Rating Scale; SD, subclinical depression; QSC, qi-stagnation constitution; fMRI, functional magnetic resonance imaging. MDD, major depression disorder.

We used the SPIRIT checklist when writing our report (25). To explore potential neuroimaging biomarkers for the progression from adolescents with SD and QSC to those with MDD, we designed a nested case-control study incorporating both longitudinal follow-up and cross-sectional comparisons. We will collect demographic data, including sex, age, and education, as well as neuropsychological assessments, such as the 60-item Chinese Medicine Constitution Scale, the Self-rating Depression Scale, and the 24-item Hamilton Depression Rating Scale (HAMD-24), from all first-year senior high school students. The 300 adolescents with SD and QSC recruited for the study will undergo rs-fMRI scans at baseline and again after one year. Participants will be stratified into two groups based on whether they progress to MDD after the one-year follow-up. Baseline rs-fMRI data will be analyzed to identify neuroimaging biomarkers that may predict the progression from SD and QSC to MDD. **Figure 1** presents a flow chart of the study.

As this is an observational study comparing neuroimaging differences between the MDD and non-MDD groups, there is no requirement for double-blinding or random assignment in this research design.

### Participants

#### Sample selection

The sample size for the present study is based on prior research, which suggests that 11.0% of adolescents aged 14 to 18 with a history of SD at baseline progress to MDD over the course of one year (26). According to power calculations for group fMRI studies (27), a minimum sample size of 18 participants per group is required to achieve a type-I error rate of 0.005. Therefore, at least 164 participants diagnosed with SD and QSC will be needed for this study. Additionally, several studies have found that psychological distress is higher among students compared to working non-student populations of the same gender and age (28). In particular, first-year senior high school students experience a period of heightened vulnerability as they adjust to new environments, which makes them relevant for this study. This protocol study will recruit 300 adolescents diagnosed with SD and QSC from all the first-year senior high school students of Zhuhai Technician College. With this sample size, the study is designed to achieve a confidence level of over 80%. All the first-year senior high school students at Zhuhai Technician College will undergo screening based on eligibility criteria.

#### Eligibility criteria

The diagnostic criteria for QSC were assessed using the Qi-Stagnation Constitution Scale, a subscale of the 60-item Chinese Medicine Constitution Scale (29). This subscale includes seven items designed to evaluate individual constitutional differences based on both physical and psychological characteristics. Each item is rated on a 5-point Likert scale, ranging from “hardly ever” (score = 1) to “almost all the time” (score = 5). The total score for the QSC is obtained by summing the scores of all items, which is then standardized to obtain the conversion score. The conversion score is calculated using the formula: Conversion Score = [(Total Score - 7)/28] * 100. A QSC diagnosis is determined by a conversion score reaching or exceeding 40, which reflects an individual’s tendency towards the condition. The subscale has been widely used in China to assess QSC and has shown good validity and reliability within the Chinese population (30).

The diagnostic criteria for SD involve all first-year senior high school students at Zhuhai Technician College first completing online surveys using the Self-rating Depression Scale (SDS). Participants who score 50 or more points on the SDS are considered potential SD cases (31). Next, the potential participants will be invited for a face-to-face interview using the HAMD-24 to confirm their eligibility, with HAMD-24 scores between ≥8 and ≤20 (32). Participants who meet these criteria but do not fulfill the diagnostic criteria for MDD, as outlined in the Fifth Edition of the Diagnostic and Statistical Manual of Mental Disorders (DSM-5), will be considered as SD.

The inclusion criteria are as follows: (1) participants who meet the diagnostic criteria for both QSC and SD (2) right-handed, aged 15-18 years old, with normal vision or corrected vision, and no history of traumatic brain injury. The exclusion criteria are as follows: (1) any bereavement; (2) bipolar disorder, major depressive disorder, anxiety disorders, and organic mental disorders; (3) a history of or family history of severe mental illnesses such as schizophrenia; (4) a history of regular medication use or use of other non-pharmacological substances affecting the central nervous system and a history of neurological disorders; (5) a history of substance abuse; (6) pregnant; (7) any contraindication for MRI (e.g., metal implant or claustrophobia). The criteria for progression to MDD are as follows: (1) reaching the diagnosis criteria for MDD according to DSM-5; (2) HAMD-24 scores >20 or SDS scores ≥ 40. The dropout criteria are as follows: (1) participants mistakenly included who do not meet the inclusion criteria; (2) participants who meet the inclusion criteria but exhibit poor compliance and voluntarily withdraw during the experiment. Specifically, all diagnoses will be confirmed by two professional TCM practitioners and psychiatrists from Guangdong Provincial Hospital of Chinese Medicine, and demographic characteristics of all participants, including age, education, and family environment (such as single-parent households and being an only child), will be collected.

### Ethics approval and consent to participate

This protocol was approved by the Ethics Committees of Guangdong Provincial Hospital of Traditional Chinese Medicine (Approval Number: ZF2023-401-01) in accordance with the ethical standards of the relevant national and institutional committees on human experimentation, and the Helsinki Declaration of 1975, as revised in 2013. Written informed consent will be obtained from all participants or their parents or legal guardians.

### Rs-fMRI image acquisition and preprocessing

Rs-fMRI scanning for all participants will be conducted using a General Electric 3.0 Tesla system. The parameters are as follows: 32 slices, repetition time (TR) = 2,000 ms, echo time (TE) = 45 ms, field of view (FOV) = 220 × 220 mm^2^, flip angle (FA) = 90°, matrix = 64 × 64, slice thickness = 4 mm, and inter-slice gap = 0.5 mm. The complete scanning session will last for 8 minutes, resulting in the acquisition of 240 volumes in total. Throughout the scanning process, participants are advised to relax, close their eyes, maintain stillness while staying awake, and avoid engaging in any specific thought processes. In addition, high-resolution T1-weighted images will be obtained for each participant with the following parameters: 188 slices, TR = 8.2 ms, TE = 3.2 ms, inversion time = 450 ms, FOV= 256 × 256 mm^2^, FA = 12°, matrix = 256 × 256, acquisition time = 250 s, and slice thickness = 1 mm without inter-slice gap.

The preprocessing of rs-fMRI data will be executed with the GRETNA toolbox (33), which is built upon the SPM12 software (http://www.fil.ion.ucl.ac.uk/spm). Initially, the first five volumes will be removed to accommodate the effects of magnetic saturation. Then, the remaining functional images will be corrected for head motion using a 6-parameter rigid-body transformation. The participants will be excluded if their images exceed 2 mm in translation or exceed 0.5 mm in mean frame-wise displacement. Subsequently, the corrected images will be co-registered to the corresponding T1 images and spatially normalized into the standard Montreal Neurological Institute space. Ultimately, the normalized images will undergo band-pass filtering (0.01 to 0.08 Hz) and removal of nuisance covariates, including 24-parameter head motion profiles (34), white matter signals, cerebrospinal fluid signals, and global signals.

### Data analysis

#### Graph-based network analysis

The functional brain networks will be constructed based on a functionally defined parcellation atlas, which divides the cortex into 400 regions of interest (ROIs) (35). The ROIs represent the network nodes, and the mean time series within each ROI will be extracted. Pearson correlation coefficient of the regional mean time series between any pairs of ROIs is calculated to measure the edge (i.e., FC) between network nodes. Then, each of the resulting correlation matrices is converted into a series of binary networks with a sparsity threshold, S, ranging from 0.1 < S < 0.4 (interval = 0.01). All network analyses will be performed at each of the threshold level. Specifically, we calculate brain network measures including graph-based global efficiency, nodal efficiency, and nodal centrality using the GRETNA toolbox (33) from the binary networks obtained. For each network measure, the area under the curve will be calculated to simplify subsequent statistical analysis (36).

#### Calculation of neuroimaging measures

We will calculate the other neuroimaging measures including ReHo and ALFF for each ROI. Briefly, we first calculate the voxel-wise ReHo and ALFF. The ReHo measures the functional synchronization of a voxel with its close neighbors (37), and ALFF measures the regional spontaneous neuronal activity (38). In our study, the ReHo value of each voxel will be denoted by the Kendall’s coefficient of concordance (KCC) of the time series of this voxel with its 26 nearest neighbors, and the ALFF value of each voxel will be obtained as the averaged square root of the power spectrum across 0.01–0.08 Hz, after transforming each preprocessed rs-fMRI dataset to the frequency domain using a fast Fourier Transform. Then, to mitigate the global effects of variability across participants, the ReHo and ALFF value of each voxel will be normalized by dividing it by the global mean ReHo and ALFF value for each subject, respectively. Finally, the individual ReHo and ALFF maps are segmented into 400 ROIs, and the mean ReHo and ALFF values of each region is acquired by averaging the ReHo and ALFF values within that region, respectively.

#### Statistical analysis

For the categorical variables, such as gender, Chi-square tests will be used. For continuous variables, including demographic data, neuropsychological test scores, and neuroimaging measures, the Lilliefors test will be firstly used to tested the normality. For demographic and neuropsychological variables, two-sided two-sample t-tests will be employed for those showing normal distributions, and nonparametric Wilcoxon rank-sum tests will be applied for the others. For neuroimaging measures, since most are expected to not follow a normal distribution, nonparametric permutation tests (10,000 permutations) will be conducted to assess between-group differences with statistical significance. The significance threshold will be set at *p* < 0.05, with corrections for multiple comparisons using the false discovery rate procedure. For FC analysis, the threshold-free network-based statistics method (39) will be employed to detect significant differences between the two groups. The significance level will be determined using a nonparametric permutation test (10,000 permutations). To control for multiple comparisons, the statistic of each edge will be compared against the distribution of the maximum statistics across all edges under the null hypothesis, ensuring robust correction for multiple testing. Specifically, age, sex, education, and family environment will be included as covariates in these analyses to account for potential confounding effects.

### Status and timeline of the study

Starting on January 1, 2024, we began recruiting participants, and by May 30, 2024, we successfully completed the recruitment of 300 adolescents diagnosed with SD and QSC, followed by conducting their fMRI scans. Over the course of the following year, we will carry out longitudinal follow-up assessments. Beginning on June 1, 2025, we will conduct a second round of clinical assessments and fMRI data collection on the 300 participants to identify those who have progressed to MDD, thereby enabling appropriate subgrouping for further analysis.

### Data management

Data will be collected prospectively and entered into electronic case report forms, which will be monitored by the trial manager and subject to auditing. Each participant will be assigned a unique study ID that will be used to identify them throughout the study. This ID will be used on all documentation and for data analysis. Any data transfers will comply with the NHS Code of Practice on Confidentiality.

## Discussion

To the best of our knowledge, this study protocol is the first nested case-control study aimed at identifying neuroimaging biomarkers that can predict the progression from adolescents with SD and QSC to those with MDD. Findings from this research will contribute valuable insights into early intervention strategies that could mitigate the progression of MDD in high-risk adolescents.

Given that both SD and QSC are important risk indicators for developing MDD in adolescents, we screen adolescents with SD and QSC and further investigate the neuroimaging biomarkers of those at high risk for MDD progression using rs-fMRI. Previous rs-fMRI studies have identified functional abnormalities in the brains of individuals with SD, and the FC alterations could distinguish SD patients from healthy controls (40, 41). In addition, Zhang et al. (2022) included patients with SD, patients with MDD, and healthy controls, and observed that increased ReHo in the bilateral precuneus was only found in MDD while increased ReHo in the right middle frontal gyrus and precentral gyrus were unique to SD (42). However, few studies reported the neuroimaging biomarkers from SD and/or QSC developed into to MDD. It’s necessary and feasible for this protocol study to compare the differences in brain rs-fMRI data between the two groups (those who progress to MDD and those who do not) to explore neuroimaging biomarkers for the progression from adolescents with SD and QSC to those with MDD.

MDD is the most common mental disorder in adolescents. Identifying neuroimaging biomarkers for the progression from adolescents with SD and QSC to those with MDD will significantly facilitate early intervention strategies. In the future, we will conduct early intervention treatments for adolescents at high risk for MDD and use cross-sectional studies to explore their neural mechanisms. This nested case-control study protocol can also provide reliable clinical and neuroimaging evidence for subsequent cross-sectional studies.

### Limitations and further considerations

The study protocol faces several limitations. First, the study may be limited by the high dropout rate among adolescents over the one-year follow-up period. This high attrition rate may result in incomplete data and reduced statistical power, affecting the reliability of the findings related to the progression from SD to MDD. To mitigate this issue, the study will implement strategies to enhance participant retention, such as providing incentives for continued participation, maintaining regular contact with participants, and offering flexibility in follow-up sessions (e.g., conducting follow-ups online or by phone). Second, the sample focused on a specific age group may limit the generalizability of the findings to adolescents in other age groups or educational stages, as psychological distress and progression to MDD could vary across different ages and developmental stages. To address this, future studies could consider broadening the sample to include adolescents from different educational levels or age groups. Additionally, conducting a multi-site study across different regions or schools could provide more diverse data and enhance the external validity of the findings, making them more applicable to a wider adolescent population.

### Dissemination

The results of the study will be submitted for peer review for publication in a scientific journal and will also be presented at national and international meetings.
